# Observations and Inquiries into the Nature and Treatment of Yellow, or Bulam Fever, in Jamaica and at Cadiz, Particularly in What Regards Its Primary Cause, and Assigned Contagious Powers :— Illustrated by Cases and Dissections, with a View to Demonstrate That It Appears Divested of Those Qualities Assigned to It by Mr. Pym, Sir James Fellowes, and Others; in a Series of Memoirs

**Published:** 1816-08

**Authors:** 


*Observations and Inquiries into the Nature and Treatment of Yellow, or Bulam Fever, in Jamaica and at Cadiz, particularly in what regards its primary Cause, and assigned contagious Powers:--Illustrated by Cases and Dissections, with a view to demonstrate that it appears divested of those Qualities assigned to it by Mr. Pym, Sir James Fellowes, and others; in a Series of Memoirs.* By EDWARD DOUGHTY, Member of the Royal College of Surgeons London, and Surgeon to the Forces. 8vo. pp. 238. London, 1816.

WE observed in a late Number of our Journal, that Government would do well to pause, ere they adopt the precautionary plans of the Contagionists. We know that very many documents of an anti-contagious nature, and from authorities of weight, have lately been forwarded, through the proper channel, to the privy council; and we have little doubt that the opinions which we have all along promulgated, will be, in the end, confirmed by a majority of evidence and facts.

Mr Amiel's paper in our Journal must have carried conviction to the unprejudiced mind, that however plausible, and apparently incontrovertible are the positions of Mr. Pym and Sir James Fellowes, they must only be viewed as *exparte* statements, till the opposite side of the question comes before the public.

We have here the publication of a gentleman who witnessed the scenes described, and whose authority is consequently as good as those of higher rank.

The first part of Mr. Doughty's work is dedicated to the yellow fever, as it appears in the West Indies, where our author served eight years, and where of course he had ample opportunity of becoming intimately acquainted with the nature of the disease. All the documents in this part of the book tend to prove, what we believe to have long been the opinion of the best practitioners in the

West Indies, that the fever termed yellow, or Bulam, is the concentrated endemic of the country, and non-contagious. His memoirs also shew the superiority of bleeding, mercurial purgatives, cold and tepid effusion, &c. over every other plan of treatment. We shall pass over this division of the work entirely, in order to extend our analysis on that more immediately interesting at the present period—the fever of Cadiz. We may just observe, however, that at page 76, Mr D. bestows approbation on a passage in a contemporary Journal, which never appeared in *that* Journal, but in ours for November last. Mr. D. has done this undesignedly, however, by the omission of a word.

Our author landed at Cadiz in July 1810, and immediately entered on duty at the *Hospicio*, and soon after at the Aguada. In the months of July, August, and September, the heat was as oppressive as Mr. D. had experienced it in the West Indies. Our author becoming extremely bilious and dyspeptic, removed for a fortnight to Gibraltar, where the temperature and sultriness of the atmosphere were still more excessive than at Cadiz. He returned to the latter place early in October, leaving Gibraltar just as the fever broke out in the transports, as stated in Mr. Pym's work.

In Cadiz it had made its debut simultaneously with that on the rock; and had excited various and discrepant opinions. The most *general* belief was, however, that the fever was of local origin. Mr. D. had little difficulty in recognising it as similar to the yellow fever of the West Indies, of which he had had such ample experience.

"I was immediately struck with the peculiar coincidence between the disease, as it then prevailed, and the yellow fever of Jamaica, in all the prominent symptoms and appearances observed."

Two officers having died of the disease in the St. Elena barracks, Mr. Doughty obtained leave to open them, and stated the result in an official letter to Sir James Fellowes. The most incontestable proofs of inflammation and congestion were found in almost all the important viscera, particularly the brain, heart, lungs, and stomach.

"It might not be unreasonable to infer, that Sir James would have *officially* acknowledged the receipt of this communication; but he never did; nor has even deigned to mention it in his publication. Perhaps it was better to be silent regarding a circumstance which might militate against the *theory of contagion.* 99.

Here we have a real specimen of the candour of the Contagionists! The medical Procustes of this Sect immediately lops off from his official returns, every document that does not lie fair and even on his bed of theory; while every fact or opinion that quadrates with his own, is *stretched* out by the pullies of hypothesis to any length or breadth that is required to form a stratum in the airy fabric or system which he is building! But the fardels of medico-military authority are thrown off; we breathe the elastic air of liberty; all ranks are now on an equal footing; and we bow the knee of opinion to no created being! The effects of this equilibrium will soon be seen; the *suppressed* evidences will rise in irresistible masses; and, like Acteon's hounds, tear every fibre of sophistry and misrepresentation from their quondam masters!

Mr. Doughty states, in the next page, "that he never had the honour of seeing him (Sir James) by the side of any one of the many bodies he opened." So strong was our Author's opinion of the fever being non-contagious, that he wrote a letter to the Duke of Kent on the subject, in which is the following passage:

"No medical officer of this army, I feel convinced, has taken such probable steps to acquire a knowledge of this disease as I have, *being the only one who has had recourse to dissection* to obtain information on this head."

This *dirty* work of *post mortem* examination was so rank an offence that it "smelled to heaven; and, what was worse, it offended the delicate olfactories of Sir James, who (reader, suppress thy indignation!) first gave an order that this prying surgeon should open no more bodies, ultimately tried him by a court-martial for the paragraph above quoted, and had him dismissed the service! "These are thy glorious works," parents and partisans of Contagion! These are the means (to wit, the bayonet and scymeter) by which, in the genuine spirit of Islamism, ye propagate the *true* faith in your contagious Koran! But the day of retribution is at hand!—On what grounds, reader, did this medical superintendant rest his prohibition of dissection? On the frivolous plea, that Mr. Doughty requested permission to open a man *before he died!*

"A request to open the body, and defer the interment of a soldier, who died of an extraordinary and complicated order of disease, but who was *not dead at the time the request was made*, from which it perhaps might and will be supposed, that I wished to *cut him up alive*, or hasten his dissolution for that purpose!" 202.

Sir James, taking hold of the above flaw, most *ingenuously* and liberally shuts the door on Morbid Anatomy!

"The Prosecutor, Sir James Fellowes, begs leave to state to the Court, that he was induced to reject the proposition (of opening the body) which had been made to him by Staff Surgeon Doughty, not only on the principle of the impropriety of making such a request, in an official shape, to the *head of the department*, but he judged it necessary to *check at once*, what bore on the face of it *a sort of inhumanity*, and a prejudicial tendency, inasmuch as it might excite in the minds of the soldiery the strongest prejudices against their medical attendants, and a belief that they were *prematurely* consigned to *death*, for the purposes of dissection and being ANATOMIZED" !!!

Heaven and Earth! what a speech from an English Physician to a Military Tribunal! No words can express our indignation; no language our contempt, for such a disingenuous and overstrained affectation of *Humanity!* The noble contrast which Mr. Doughty's own conduct and expressions afford, will be the best means of consigning the above passage to the execration of every liberal mind, and every lover of truth.

"It was my custom, when I proceeded to open the body of a deceased soldier, to address the orderlies or others who might be in attendance to assist me, thus: '*It is my wish, soldiers, from what I am about to do, to find out, if I can, why this poor man died, and thereby, if possible, prevent you or others, who may labour under a similar disease, from falling victims to it also.*' They always listened to me with great attention, and took particular interest in what they saw, exhibiting no signs of alarm or apprehension"

No, certainly; the "alarm and apprehension" existed only in the mind of the Superintendant.—But, to return. Previously to this medical *inquisition* on the sins of anatomy, Mr. Doughty was enabled to examine the bodies of many victims to this fever; and eight dissections, with cases more or less detailed, are given; from which

"I appeal (says Mr. D.) to every unbiassed Reader, who may honour these pages with a perusal, or who has seen Yellow Fever in any one of the West India Islands, whether the shades of difference in the several symptoms, during the progress of the disease, or the morbid appearances after dissolution, can any ways be said to constitute a distinct order of fever from that which *ever has* been annually, more or less, and I fear, ever will be, amongst strangers from the more northern regions: a periodical visitant of intertropical climates, in particular situations; and an occasional attendant in certain places lying in or near the 40th degree of latitude." 171.

These *post mortem* appearances would certainly, in any unprejudiced mind, have indicated the use of the lancet; but no such weapon was ever wielded against this formidable endemic — another consequence of the contagion doctrine! In our review of Sir James's Reports, we stated that there appeared ample materials for originating a fever in the "*Barrio or district of Sante Maria,*" without any imported fomites from the western hemisphere. Our opinions are confirmed by an eye-witness, who took up his residence in the very vicinity of this focus of disease.

"The olfactory nerves (says Mr. D.) were here assailed with the most noxious exhalations, and the eyes disgusted with every sort of filthy and excrementitious matters thrown indiscriminately into the streets. Fish bones, rotten vegetables, and rotten matters of every description, mixed together by contents from the receptacles of the night, formed the *delectable* covering of most of those extremely crouded and ill ventilated streets. Will any one tell me, that if Cadiz was built on a rock of adamant, and its streets to be covered from time to time with matters of this description, on which the solar influence might operate a degree of heat equal to 95, or 100, often experienced out of the shade in that city, in the summer months, there would not be just grounds to expect the generation of fever." 180

We answer for ourselves, that we never entertained any doubt on the subject, and that we stated this opinion in our Journal for November, 1815, page 404.

In respect to Mr. Pym's grand argument, namely, the *non-liability* to second attacks, Mr. Doughty acknowledges that persons who have once passed through the more aggravated forms of endemic fever, are comparatively secure, *for a time,* from subsequent attacks, and indeed this may be said of typhus, and the plague itself. But we are convinced with Mr. Doughty that this immunity is only temporary, till the susceptibility is again regenerated by a colder climate, or purer air in the same climate.

Mr. D. here instances the case of the 85th Regiment, which suffered dreadfully from the concentrated form of yellow fever in Spanish Town in 1805. The next season they escaped it entirely in Fort Augusta, but again in 1807 they were nearly annihilated in Kingston. At the latter time and place, Mr. Doughty himself was at the brink of the grave from an attack of yellow fever, though seven years previously, he asserts that he had it in its concentrated form in the same town! How will Mr. Pym explain away these facts? By a few dashes of the pen.

Mr. Pym rests much of his force on the immunity which certain individuals whom he specifies, experienced on the rock of Gibraltar, because they had had the fever in the West Indies. We could wish him to explain the following immunity:

"In the season of 1810, (at Cadiz) to which my own particular observations relate, there were doing duty within the walls of Cadiz, and at the Aguada, two physicians to the forces, two surgeons to the forces, one deputy purveyor, an apothecary, five or six clerks, and twelve or fourteen regimental and general hospital assistants, not one of whom (independently of Sir James Fellowes and myself) *had ever been in the West Indies, or where this fever before prevailed;* yet not one of them was attacked, although several attended patients labouring under the disease, as well as assisted me in the dissections I have given." 192. And a little further on: — "Reason, calm deliberate reason, will, ere long, remove the veil of prejudice which the idea of contagion has thrown over the human mind in regard to this fever, and exhibit to impartial discrimination, the fallacy of the doctrine. A doctrine highly prejudicial to humanity, as well as to the various commercial and other pursuits in life. A cause which would not have in itself a power to extend but to few, may, by the debilitating influence of fear, and apprehension of contagion, become general in its effects, from the highly increased state of susceptibility to its influence which that fear and apprehension wouldcertainly create. I told the gentleman who assisted me in the dissections, and I told the orderlies who lifted the bodies to and from the table that they had nothing to fear — that what they saw or smelt was harmless. They believed me; and it proved so." 194.

These are stubborn facts in favour of non-contagion, and are seriously recommended to the consideration of the legislature.

The difference of medical topography in Cadiz and the *Isle de Leon* is very striking, and satisfactorily accounts for the superior salubrity of the latter. The town of *Isla* is formed principally of one long, wide street, the buildings of which are large and not crowded together. They are consequently well ventilated and free from filth. The *Isla* in its soil is almost entirely marine sand, and is washed in three-fourths of its limits by the sea. The neighbouring *marshes,* as they are called, are mere excavations of the earth for the reception of sea water, and are perfectly barren, or sprinkled here and there with the barilla shrub. It is evident that they are not the sort of marshes which exhale febrific miasmata. We would now wish to turn the attention of the medical public towards the *treatment* of the Cadiz epidemic in 1810, which flowed from the *Bulam* theory.

"In not one case of the fever at Cadiz in 1810, was venæsection ventured on; how far its utility in the early stage of that disease, would have been found, I can therefore only conjecture. The morbid appearances discovered on dissection, particularly the striking indications of strong vascular action in the brain, with congestions and extravasations of blood; the same in the lungs and thoracic cavities; and the almost invariable appearance of inflammation of the villous membrane of the stomach, clearly, I think demonstrate, that a copious abstraction of that fluid, at the onset of the fever, would have been a judicious practice; and that those derangements found in the parts mentioned, would have been thereby averted. Hence, the great utility of inquiry by dissection, when our endeavours in the treatment of an insidious disease have proved abortive." p. 218.

It is melancholy to think that, after all the lights which have been thrown on the subject of fever, and many other diseases of late years, and the almost innumerable evidences of the utility of blood-letting, and other evacuations therein, an hypothetical preconception should, in 1810, give rise to a mode of treatment little better than that which resulted from the ravings of Brunonianism!

In order to substantiate our charge, we shall here give as a specimen of the general practice, one of the cases detailed by Mr. Doughty, with the appearances on dissection.

"Mr. Bower, ætat. 41, was attacked with fever on the 19th of November, accompanied by violent vomiting and purging, the matters ejected being bitter to the taste. Cathartic extract and calomel till copious evacuations were procured.

"20th. In the evening Dr. Plenderleath was called in, who considered it a well marked case of the epidemic. His countenance at this time exhibited a very *flushed appearance;* he had much pain across the *forehead*, and great sickness at stomach. Tongue white and dry; pulse rather full, *strong*, and *expanding;* frequent bitter vomiting. Dr. P. directed two grains of calomel, two of antimonial powder, and half a grain of *opium* every *two hours.* (!) Four ounces of infus. sennæ were ordered, but not given. The pills were continued all day, and a purgative enema administered 9 P. M. Symptoms the same. The injection repeated. Dr. P. ordered a blister to the stomach, and one grain of *opium* to be taken. (! ! !)

"21st. Dr. P. considered his patient had a remission of fever, and directed from one to two drachms of bark in substance to be given every three hours; the above-mentioned pills to be given alternately."

"22d. This morning, Dr. P. considered his patient in a state favourable to recovery. In the *evening* he observed, that a most unfavourable *change* had taken place." (No wonder!) "The nausea and vomiting recurred;* a diffused yellowness appeared over the surface of the body. Stupor. Blister to the nape of the neck; purgative injection. Tincture of bark and sulphuric æther to allay the vomiting. One grain of opium at bed-time.

"23d. Patient thinks himself better; pulse not very unnatural. Tongue black towards the uvula, but moist; yellow suffusion of a darker hue, particularly about the neck and breast. 12 *o'clock.* Slumbering and groaning alternately; insensible, except when roused; tongue black, dry, and rough. He expired at ten o'clock at night.

"*Sectio cadaveris.* Surface of the body exhibited a light yellow suffusion intermixed with livid blotches, as in the other cases. On removing the skull-cap, a considerable quantity of *extravasated* fluid blood in the direction of the falx. The whole surface of the cerebrum in a *great degree inflamed.* In several parts, bright red spots, and over the pia mater generally, a white lymphy appearance. Great vascular turgidity throughout the several convolutions of the brain, from the substance of which, when cut into, a considerable portion of red bloody fluid escaped. Anterior lobes of the brain resting on the optics greatly inflamed, and the vessels distended. About a drachm of yellow fluid in each ventricle; interior surface of the ventricles very vascular; plexus choroides uncommonly so. On removing the cerebrum, a portion of extravasated blood was found in each depression of the skull. Surface of the cerebellum inflamed, and covered with a lymphy matter; about an ounce of a sanious fluid round the medulla oblongta; medullary substance of cerebellum dotted with red particles of blood. In the thorax nothing very unusual. In the stomach about half a pint of a greyish fœtid fluid; various parts of the villous membrane of this viscus exhibited an appearance like matter of this colour, and in various parts had evident marks of great inflammation, particularly about the fundus. Spleen rather large, and black internally; liver very large, but structure not apparently altered, except on the concave surface of the great lobe, where it was of an olive colour. Gall-bladder containing inspissated bile like tar" p. 132.

In our Reviews of the different writings on the present subject, we have distinctly stated, that we were of neither party; we differ in opinion both from the contagionists and non-contagionists; and we are not personally acquainted with any individual on either side. Any warmth of expression, then, which may escape us, cannot be attributed to *personal* or *party* motives, but to our ardent desire for truth and fair representation. We now confidently appeal to the profession at large, whether the

* If the reader will compare this case and treatment with those detailed under the head "*Endemic of Batavia,*" in Mr. Johnson's work, *ten years* previously, he will see a melancholy *similarity;* and an incontestible proof, that the *Bulam* contagion produced the same ravages on the brain of medical men in Edam and Cadiz!

documents here brought forward, if authentic, (and we have no reason to doubt their authenticity) do not prove the practice at Cadiz in 1810 to be, if possible, more injudicious than that which was so fatally exemplified at Batavia ten years before. With these dissections *officially* before the Head of the Medical Department, whatever his rank, we ask the profession, whether the exhibition of opium, and the omission of blood-letting, were not most admirably calculated to augment those congestions and inflammations which destroyed the function and structure of the viscera above-mentioned? and, consequently, whether the injudicious, though doubtless well meant, interference of Art, did not produce a result less felicitous, than if the sufferers had been left to the efforts of unassisted Nature?

It behoves Sir James Fellowes, and every physician and surgeon concerned in this scene of sickness, to justify their conduct on the occasion, by stating to their brethren and the world, their reasons for neglecting to ascertain the morbid effects of the fever, or for shutting their eyes against the researches of their fellow practitioner. We would recommend them not to contemn public opinion on this occasion; for, assuredly, as far as *ex parte* evidence goes, the current is strongly against them.

We shall conclude this Review with one more Extract.

"If the Parliament of England was to reverse the order of its wisdom and its liberality, in rewarding evil for good, and by way of experiment, offer a large premium for the introduction of yellow fever into any part of Great Britain, either from the West Indies or from any of the ports of Spain, I am well assured, that all attempts would prove abortive. It cannot, I repeat, be propagated in a soil which does not in itself, impart the seeds of the disease." p. 215.

Notwithstanding some few verbal inaccuracies, and inattention to arrangement, the volume before us bears the character of respectability for composition; while the matter and manner convince us that Mr. Doughty possesses, in an eminent degree, that professional *enthusiasm* which, in spite of "the oppressor's wrong, and proud man's contumely," will one day recompence him for the toils and labours he has undergone through zeal for the cause of science and humanity.

